# Sending Plants to Sleep

**Published:** 1877-03

**Authors:** 


### Sending Plants to Sleep.

Several members of the Parisian Biological Society have recently been engaged in a series of experiments which seem to prove that everything endowed with life, whether animal, plant, or ferment, is susceptible of being brought under the influence of anæsthetics —in other words, may be sent to sleep. It has been proven that the influence of anæsthetics extends to all the animal tissues, and last of all to the central nervous system. Hence, it was argued, plants having tissues must also be subject to the influence of ether, etc. Experiments prove this to be the case. Germination is arrested by anæsthetics. The water-cress, for example, germinates within thirty hours. Ether arrests germination in this plant, but does not destroy that faculty. It merely sends the plant to sleep, for germination re-commences as soon as the use of ether is suspended. But the sensitive plant furnishes a still more striking illustration. Its sensitive faculty is rendered completely dormant by etherization, while the other living properties remain unaffected. On suspending the action of ether, the sensitive faculty of the plant is quickly restored. This capability of being sent to sleep is not confined to plants; it extends to ferments. Thus the ferment of beer, when submitted for twenty-four hours to the influence of ether, becomes perfectly dormant, but recovers its activity as soon as the anæsthetic action is suspended. In future the practical botanist must not pursue his cruel rambles without the assistance of one of the Clover family.—*Med. Exam.*

